# Double spinal dural arteriovenous fistula with multiple draining veins: A case report

**DOI:** 10.1097/MD.0000000000043853

**Published:** 2025-08-08

**Authors:** Rui Shang, Jin Li, Yu-Hu Ma, Ting Wang, Hai-Tao Hu, Seidu A. Richard, Chang-Wei Zhang

**Affiliations:** a Department of Neurosurgery, West China Hospital, Sichuan University, Chengdu, Sichuan Province, P.R. China; b Institute of Neuroscience, Third Affiliated Hospital, Zhengzhou University, Zhengzhou, Henan Province, P.R. China; c Department of Biochemistry and Forensic Sciences, School of Chemical and Biochemical Sciences, C.K. Tedam University of Technology and Applied Sciences (CKT-UTAS), Navrongo, Ghana.

**Keywords:** angiography, endovascular, hybrid theater, SDAVF, surgery, synchronous

## Abstract

**Rationale:**

Spinal dural arteriovenous fistulas (SDAVFs) are acquired arteriovenous shunts that occurs in the layers of the dura. Double-level SDAVFs with multiple draining veins are extremely rare. We present a rare case of double-level SDAVFs with multiple draining veins which was successfully managed in our hybrid theater.

**Patient concerns:**

A 54-year-old male presented with 6-week progressive bilateral lower extremity paresis accompanied by urinary incontinence.

**Diagnoses:**

Selective spinal angiography revealed multiple DAVFs at T6 and T9.

**Interventions:**

Surgery was performed in our hybrid operating room because of complexity of both fistulas. Intraoperatively, angiography was performed to identify the SDAVFs prior to electrocoagulation and removed.

**Outcomes:**

The patient recovered with no further neurological deficits and 2 years follow-up revealed the patent is well and go about his daily duties.

**Lessons:**

Intraoperative angiography is very helpful in identify the SDAVFs prior to electrocoagulation and removed.

## 1. Introduction

Spinal dural arteriovenous fistulas (SDAVFs) are acquired arteriovenous shunts that occurs in the layers of the dura.^[1,2]^ They constitute for about 75% to 80% of all spinal vascular malformations.^[1]^ Pathologically, SDAVFs can occurs as single or multiple lesions.^[1,3]^ The multiple SDAVFs are classified into types such as the synchronous in which 2 or more fistulas occur concurrently and metachronous inch which the fistulas occur at different times.^[3]^ Double-level SDAVFs with multiple draining veins are extremely rare and they account for only 1.4% of all SDAVFs.^[1,4]^

Double SDAVFs are typically 2 lesions found at a single or double nerve root, resulting in progressive spinal venous hypertension, venous drainage as well as neurological dysfunction such as paraparesis, sensory disorders as well as bowel and bladder dysfunction.^[5]^ Computed tomography angiography or magnetic resonance angiography are capable of identifying the shunt site. Open surgical procedures or endovascular therapies are the 2 main treatment modalities for SDAVFs.^[1,6]^ It is very important to conduct spinal angiography to identify the shunt location and analyze the vasculature prior treatment.^[7]^ We present a rare case of double-level SDAVFs with multiple draining veins.

## 2. Case report

A 54-year-old male was admitted at Department of Neurosurgery, West China Hospital with 6-week progressive bilateral lower extremity paresis accompanied by urinary incontinence 10 days prior to admission. He is a local farmer. He has no past history of tuberculosis, hypertension, and diabetics. Physical examination disclosed hypesthesia of the perineum which extended to lower limbs, proprioceptive sensory loss in the lower limbs, amplified deep tendon reflexes, bilateral lower extremity weakness (muscles power of 1/5 in both limbs), as well as urinary incontinence. Routine laboratory investigations were at normal ranges and electrocardiogram as well as chest X-ray did not show any abnormities.

**Figure 1.**
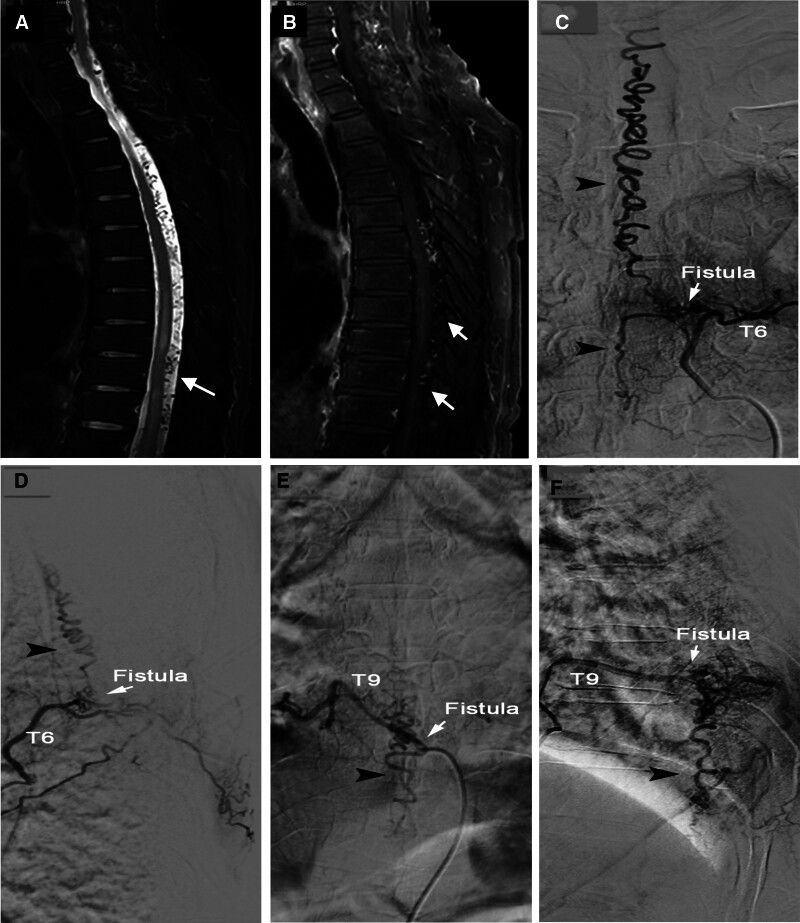
Preoperative spinal T1 and T2 weighted magnetic resonance imaging (MRI) and angiography. (A) A T2 weighted image showing spinal cord edema and tortuous vessels on the surface of the spinal cord (white arrow). (B) A T1 weighted image showing spinal cord edema and tortuous vessels on the surface of the spinal cord (white arrow). (C, D) The frontal and lateral views of the T6 spinal dural arteriovenous fistula (white arrow) and draining vein (black arrow). (E, F) The frontal and lateral views of the T9 spinal dural arteriovenous fistula (white arrow) and draining vein (black arrow).

Spinal T1 and T2 weighted magnetic resonance imaging (MRI) revealed mild swelling of the spinal cord at T8 to T9 and the conus medullaris, as well as multiple tortuous and thickened vascular structures surrounding the spinal cord (Fig. 1A and B). Base on the finding above a diagnosis of vascular malformation was made. However, selective spinal angiography revealed multiple DAVFs at T6 and T9. Two fistulas were found lateral to the dural, and the drainage veins were tortuous and thickened, surrounding the spinal cord surface (Fig. 1C–F). The DAVF located at T6 mainly drains to upper veins as well as some draining veins below. The DAVF located at T9 mainly drains downward. Also, both fistulas were located at posterior side of the spinal cord. According to the imaging findings and patient symptoms, surgical treatment was scheduled for patient. The surgical plan was resection of T6 and T9 spinal DAVF, nerve root decompression, and lamina reconstruction.

**Figure 2.**
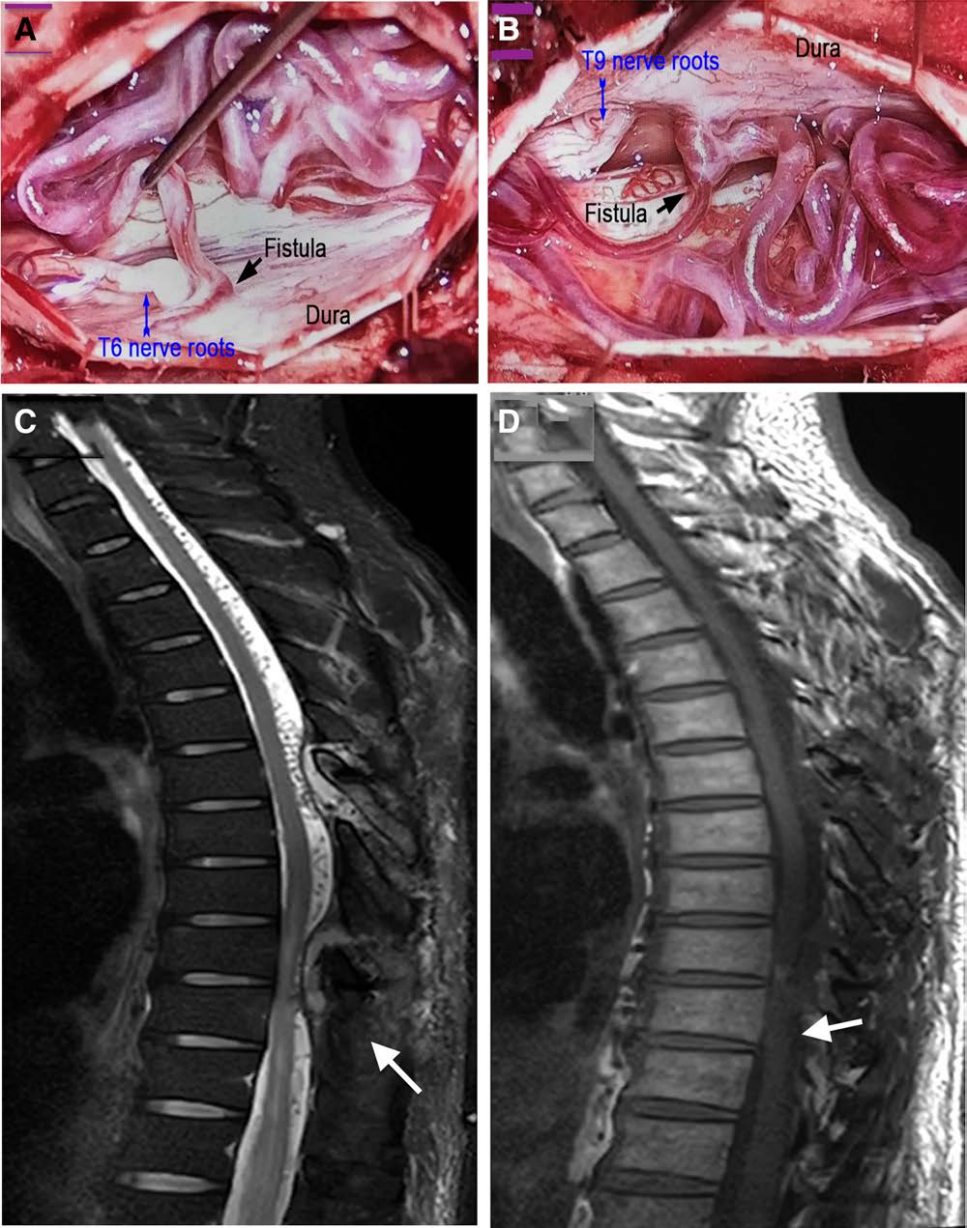
Intraoperative images and postoperative spinal magnetic resonance imaging (MRI). (A, B) Fistulas (black arrows) and draining veins around the T6 and T9 nerve roots that were successfully excised. (C) A T2 weighted image showing disappearance of spinal cord edema and superficial tortuous vascular shadows on MRI after surgery (white arrow). (D) A T2 weighted image showing disappearance of spinal cord edema and superficial tortuous vascular shadows on MRI after surgery (white arrow).

The surgery was performed in our hybrid operating room because of complexity of both fistulas. After satisfactory anesthesia, the patient was in a prone position. C-arm was used to re-affirm the position of lesions and disinfection carried out. The skin was incised along T6 to T9, the muscles were separated, and the Medtronic power system was used with the lamina rongeurs to remove the lamina, and the T6 and T9 lamina were incised from the back. The dura mater was opened, and tortuous and thickened veins as well as the T6 and T9 DAVFs were seen were seen on the surface of the spinal cord (Fig. 2A and B). The nerve roots were separated to allow for sufficient decompression. After angiography, the leakages from T6 and T9 DAVFs were confirmed, and the DAVFs were electrocoagulated and removed. Complete hemostasis, repair of the dura mater, insertion of the bone flap for lamina reconstruction, placement of drainage tube, and suturing of the muscles, fascia, and skin in layers was carried.

The patient was sent to the anesthesia recovery room after the operation. Postoperative course was uneventful and his low limbs symptomatology and urinary incontinence resolved. His wound healing progressed normally with no neurological deficits. Postoperative T1 and T2 weighted MRI showed that spinal cord edema was improved (Fig. 2C and D) and the patient was discharged 2 weeks after the operation. The patient recovered with no further neurological deficits and 2 years follow-up revealed the patent is well and go about his daily duties.

## 3. Discussion

Multiple SDAVFs are rare and most reported cases were identified either synchronously or metachronously during reevaluation of the patients.^[7–9]^ Double SDAVFs are rare and the account for about 1% to 2%.^[7]^ Notably, although the precise pathophysiology of DAVFs remains uncertain, late onset of symptomatology, lack of other related vascular anomalies, prevalence in males as well as development of fistulas following trauma or surgery points towards an acquired etiology.^[7,10]^ It has been speculated that 1 SDAVF could trigger occurrence of another SDAVF due to elevated pressure, venous stagnation, as well as subsequent thrombosis.^[9]^

The literature on double SDAVFs show that in a series of 56 patients, Merland et al identified 4 patients with double SDAVFs^[11]^ while Pierot et al identified 2 patients in a series of 50 patients.^[9]^ Also, van Dijk et al identified 1 patient in a series of 49,^[12]^ while Krings et al identified 1 patient in a series of 129 patients.^[6]^ Therefore, our case is worth reporting due to the rare nature of double SDAVFs in literature. The symptomology of double SDAVFs due to progressive spinal venous hypertension, venous drainage as well as neurological dysfunction such as paraparesis, sensory disorders as well as bowel and bladder dysfunction.^[5]^ Our patient presented with progressive bilateral lower extremity paresis which was accompanied by urinary incontinence.

MRI is often unable to establish whether there is fistula or not, thus, it is unable determine whether the fistula is single or double SDAVF.^[6,7]^ It is only able detect abnormal veins of drainage and the location of these veins may determine the level of the SDAVF.^[7]^ However, these veins often spread along the whole length of the spinal cord.^[7]^ It is worth noting that the detection of all double SDAVFs was established on angiography.^[6]^ In our patient, angiography was used to establish the diagnosis because MRI only showed mild swelling of the spinal cord at T8 to T9 and the conus medullaris, as well as multiple tortuous and thickened vascular structures surrounding the spinal cord which was consistent with the diagnosis of vascular malformation.

It worth noting that localization of lesions using magnetic resonance angiography or computed tomography angiography before spinal angiography is often helpful in the diagnose as well as treat multiple remote SDAVFs.^[1,7]^ However, angiography is the gold standard for the diagnosis of double SDAVFs because, it able to establish the diagnosis as well as characteristics the SDAVF before treatment. It able to establish the level of the fistula, it is location such as anterior or posterior in the spinal cord, venous drainage, as well as segregation of normal spinal vasculature.^[1,13]^ SDAVFs can be localized using intercostal, cervical, lumbar, sacral, as well as intracranial arteries.^[14]^

Nevertheless, whole-spinal angiography is associated with percentage risk of permanent as well as transient neurological deficits of 0.2% to 1.0% and 4.6%, respectively.^[15]^ Thus, whole-spinal angiography is not routinely necessitated due to the exceedingly low incidence of multiple SDAVFs.^[4]^ Also, multiple SDAVFs are mostly seem within a range of 3 or fewer vertebral levels.^[16]^ Thus, further injections into the adjacent segmental arteries around the recognized fistula zone are optional upon discovery of an SDAVF. Furthermore, localizing the fistulas prior to spinal angiography is essential in treatment of multiple remote SDAVFs.^[7]^

It is worth noting that treatment of both single and multiple SDAVFs are obligatory because they often lead to progressive clinical deterioration as a result of progressive occlusion of the normal radicular veins draining the medullary veins.^[7,17]^ Also, delayed treatment often decreases the possibilities of clinical recovery.^[18]^ Occluding the primary draining vein is often the goal of treatment. This achievable via either surgery, embolization therapy or a combination of both surgery and embolization therapy.^[19]^ In our patient, the surgery was performed in our hybrid operating room utilizing both surgery and angiography because both fistulas were located at posterior side of the spinal cord. Intraoperatively, angiography was used locate the leakages from T6 and T9 DAVFs and the DAVFs were electrocoagulated and removed.

Embolization therapy is a more preferable primary treatment modality due to its minimal invasiveness.^[7,20]^ Also, embolization therapy seems preferable to surgery in patients with difficult surgical location such as lesion located at the upper thoracic level or lesion located anterior to the spinal cord.^[7]^ On the other hand, surgery is preferable to embolization therapy in patients with neighboring normal spinal arteries that are likely to occluded during injection of glue.^[7,20]^ Recurrence is prevented in embolization therapy if the glue extends to the proximal portion of the draining vein.^[20]^ It worth noting that embolization therapy or surgery stands a 15% to 20% risk of recurrence due to inadequate treatment.^[20]^

Initial study observed an overall cure rate of about 50% and an improvement rate of about 30% after hybrid surgery.^[21]^ Sun et al in cohort of 45 patients observed an overall improvement rate of 77.8%, with 17.8% of patients demonstrating no change, indicating the positive role of the hybrid surgery platform for SDAVF.^[22]^ Thus, they achieved overall favorable result. In terms of complications, 2 of their patients had loss of muscle strength in both lower limbs as well as bladder sphincter dysfunction as a result of spinal cord edema, which did not improve appreciably after 6 months of neurological rehabilitation. Therefore, hybrid surgery offers a positive value of pain relief, recovery of spinal cord function, decrease anxiety as well as depression, and improvement in independent living status with very minimal complication rate.^[22]^

## 4. Conclusion

Double-level SDAVF with multiple draining veins is a very rare occurrence. Hybrid operating room was crucial in the treatment of the patient because we utilized both surgery and angiography to successfully treat the patient. Intraoperative angiography is very helpful in identify the SDAVFs prior to electrocoagulation and removed.

## Author contributions

**Conceptualization:** Rui Shang, Jin Li, Ting Wang, Hai-Tao Hu, Seidu A. Richard, Chang-Wei Zhang.

**Data curation:** Rui Shang, Jin Li, Yu-Hu Ma, Ting Wang, Hai-Tao Hu, Seidu A. Richard, Chang-Wei Zhang.

**Formal analysis:** Rui Shang, Yu-Hu Ma, Ting Wang, Hai-Tao Hu, Seidu A. Richard.

**Investigation:** Rui Shang, Jin Li, Yu-Hu Ma, Ting Wang, Hai-Tao Hu, Seidu A. Richard, Chang-Wei Zhang.

**Methodology:** Rui Shang, Jin Li, Yu-Hu Ma, Ting Wang, Hai-Tao Hu, Seidu A. Richard, Chang-Wei Zhang.

**Supervision:** Chang-Wei Zhang.

**Writing – original draft:** Rui Shang, Seidu A. Richard.

**Writing – review & editing:** Rui Shang, Jin Li, Yu-Hu Ma, Ting Wang, Hai-Tao Hu, Seidu A. Richard, Chang-Wei Zhang.
